# Rethinking lymphodepleting preconditioning in adoptive T cell therapy for durable tumor control

**DOI:** 10.1016/j.omton.2026.201293

**Published:** 2026-08-01

**Authors:** Diego Figueroa, Vincenzo Borgna, Alvaro Lladser

**Affiliations:** 1 Centro Basal Ciencia & Vida, Fundación Ciencia & Vida, Santiago, Chile; 2 Facultad de Medicina, Universidad San Sebastián, Santiago, Chile; 3 Servicio de Urología, Hospital Barros Luco Trudeau, Santiago, Chile

## Main text

Adoptive T cell therapy (ACT) has transformed the treatment of several hematologic malignancies, yet patients may develop resistance and disease come back. Moreover, durable responses in solid tumors remain by far challenging. A central goal in the field has been to improve the engraftment, expansion, and persistence of the infused T cells. For this reason, non-myeloablative lymphodepleting chemotherapy is incorporated into most ACT protocols to create “space” and cytokine availability for transferred cells by removing competing immune cells. However, this strategy might also be eliminating something from the host immune compartment that ACT may need for durable tumor control.^1^^,^^2^^,^^3^

In our recent study published in Nature Communications, we unveiled a new mechanism how transferred CD8^+^ T cells cooperate with endogenous antitumor immunity in solid tumors, and we asked how this cooperation is affected by lymphodepleting preconditioning.^4^ We used murine TCR-based ACT models in which tumor-specific CD8^+^ T cells were adoptively transferred into mice bearing established tumors expressing the cognate target antigen. Optimal ACT eliminated established tumors and promoted long-term survival. During active tumor rejection, we observed that the response was not limited to the transferred cells. ACT also expanded host CD8^+^ T cells in tumors and draining lymph nodes, including PD-1^+^GzmB^−^ and PD-1^+^GzmB^+^ populations with tumor-reactive, progenitor/exhausted, and tissue-resident features.

We next tested whether these host cells were bystanders or contributors of tumor control. Several complementary experiments pointed to an essential role for the host immune system. ACT was ineffective in RAG1-deficient mice, which lack mature T and B cells, or blocking lymphocyte egress with FTY720 prevented the accumulation of host T cells in tumors and impaired tumor control. Most directly, depletion of host CD8^+^ T cells before ACT abolished efficient tumor eradication and the associated survival benefit, indicating that host CD8^+^ T cells were the critical effector population during the initial response.^4^

We then asked how transferred cells promote the host CD8^+^ T cell response. We focused on tumor necrosis factor (TNF), an effector cytokine produced by activated T cells that can shape dendritic cell function. ACT increased activation of conventional type 1 dendritic cells (cDC1s) in tumors and migration to draining lymph nodes. These effects were lost when we blocked TNF or transferred TNF-deficient T cells, abrogating host PD-1^+^ CD8^+^ T cell expansion and impairing durable tumor control. Besides, depleting cDC1s using Langerin-DTR mice similarly prevented host CD8^+^ T cell expansion and converted an otherwise curative ACT response into a transient antitumor effect. Together, these experiments showed that transferred cells, through TNF, activate cDC1s, which then prime and expand endogenous CD8^+^ T cells, helping to propagate a second wave of antitumor immunity.^4^^,^^5^

This host response was particularly important when we modeled antigen escape. Loss of the targeted antigen is a major mechanism of resistance to T cell therapies, especially in heterogeneous tumors.^6^ We therefore rechallenged mice that had cleared primary B16F10-OTI tumors (expressing the OTI model antigen) with wild-type ACT-resistant B16F10 cells lacking the targeted antigen OTI. Most ACT-cured mice rejected this antigen-negative rechallenge. Protection was lost when we depleted CD8^+^ T cells before rechallenge or when the initial ACT used TNF-deficient transferred cells. These data indicated that ACT had promoted antigen spreading: the endogenous immune system learned to recognize additional tumor antigens beyond the ACT target. Interestingly, CD4^+^ T cells, although dispensable for primary tumor rejection, were required for this durable protective immunity, consistent with their role in supporting memory CD8^+^ T cell responses.^4^^,^^7^

We then turned to lymphodepleting preconditioning. Cyclophosphamide produced the expected acute immune depletion and expansion of transferred T cells, but its impact on endogenous antitumor immunity was prolonged. Even weeks after treatment, tumors had reduced immune infiltration and sustained loss of host CD8^+^ T cells and cDC1s, while draining lymph nodes showed incomplete recovery of CD8^+^ T cells and cDC1s and a near absence of PD-1^+^ host CD8^+^ T cells. Thus, lymphodepletion did not simply create a transient window for transferred cell engraftment; it weakened host immune landscape.^4^

**Figure 1:**
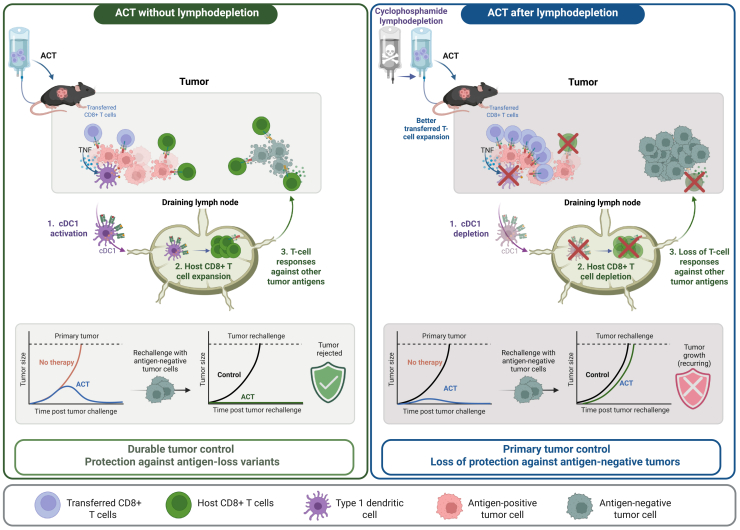
Lymphodepleting preconditioning enhances transferred T cell expansion but compromises ACT-induced durable host antitumor immunity Adoptive T cell therapy promotes early tumor control through transferred CD8^+^ T cells and, in parallel, induces TNF-dependent activation of conventional type 1 dendritic cells. Activated cDC1s support the expansion of host CD8^+^ T cells in the draining lymph node and promote T cell responses against other tumor antigens through antigen spreading. This broadened endogenous immune response contributes to durable tumor control and protects against rechallenge with antigen-negative tumor variants. In contrast, cyclophosphamide-mediated lymphodepletion improves the expansion of transferred CD8^+^ T cells and favors early control of the primary antigen-positive tumor, but depletes cDC1 and host CD8^+^ T cells, impairing endogenous T cell priming, T cell responses against other tumor antigens, and long-term protection against antigen-loss variants. This model highlights a trade-off of lymphodepleting preconditioning in ACT: enhanced acute transferred T cell activity at the cost of weakened host antitumor immunity and increased susceptibility to tumor recurrence.

This created a striking trade-off. Cyclophosphamide enhanced expansion of transferred T cells and improved control of primary antigen-positive tumors, even converting a suboptimal ACT dose into an effective therapy. However, mice that received cyclophosphamide before ACT failed to reject wild-type antigen-negative tumor rechallenge. We observed the same principle using a second model, MC38-PMEL colorectal tumors: lymphodepletion abolished long-term protection against ACT-resistant tumor cells lacking the target antigen. Low-dose cyclophosphamide also compromised durable protection. These results suggest that preconditioning can improve the acute performance of the infused T cells while undermining the host immune response needed to control antigen-loss variants.^4^ These contrasting effects of ACT with and without lymphodepleting preconditioning are summarized in Figure 1.

To explore whether this biology might be relevant in patients, we analyzed published melanoma ACT datasets. In pretreatment bulk RNA sequencing (RNA-seq) from patients receiving TIL therapy, gene signatures associated with progenitor-exhausted CD8^+^ T cells, terminally exhausted/cytotoxic CD8^+^ T cells, cDC1s, and TNF signaling were enriched in clinical responders. Single-cell RNA-seq data further showed that responding tumors contained expanded Tpex and Tex populations, along with higher TNF signaling and activation scores in cDC1s. In TCGA melanoma, high expression of these same signatures also correlated with improved overall survival. These analyses of human data are correlative, but they align with our mouse data and support the idea that effective ACT may depend on an active CD8^+^ T cell-cDC1 network within the host.^4^^,^^8^^,^^9^

Our findings suggest that ACT should not be viewed solely as a strategy to replace ineffective endogenous immunity with more potent transferred cells. Instead, transferred T cells can function as catalysts that reinvigorate host antitumor immunity. This distinction matters because durable tumor control, especially in antigenically heterogeneous solid tumors, may require both compartments: transferred cells for rapid debulking of antigen-positive disease and host CD8^+^ T cells for broader antigen coverage and long-term surveillance.

Clinically, our work suggests that the field should reevaluate preconditioning regimens that preserve host cDC1 and CD8^+^ T cell immunity. This may become particularly important as cell products with greater intrinsic stemness enter the clinic. In a recent first-in-human study, donor-derived CAR TSCM cells expanded and persisted better than standard CAR T cells and achieved complete responses at low doses without lymphodepletion, suggesting that superior cell-intrinsic fitness can reduce dependence on preconditioning.^10^ Although shown in a hematologic setting, this provides proof of principle for lymphodepletion-sparing ACT strategies that preserve host immunity while maintaining potent antitumor activity.

## Acknowledgments

This work was supported by the Centro Basal Ciencia & Vida, FB210008 from ANID – Agencia Nacional de Investigación y Desarrollo (A.L. and V.B.); FONDECYT grants 1212070 and 1251312 (A.L.), 11190897 (V.B.), and 3240458 (D.F.) from ANID – Agencia Nacional de Investigación y Desarrollo; and a PhD Scholarship 21180968 (D.F.) from ANID – Agencia Nacional de Investigación y Desarrollo.

## Declaration of interests

The authors declare no competing interests.

## Declaration of generative AI and AI-assisted technologies in the writing process

During the preparation of this work, the author(s) used ChatGPT/OpenAI in order to assist with language editing and grammar correction. After using this tool/service, the author(s) reviewed and edited the content as needed and take(s) full responsibility for the content of the published article.

## References

[1] Rosenberg S.A., Restifo N.P. (2015). Adoptive cell transfer as personalized immunotherapy for human cancer. Science.

[2] Dudley M.E., Wunderlich J.R., Yang J.C., Sherry R.M., Topalian S.L., Restifo N.P., Royal R.E., Kammula U., White D.E., Mavroukakis S.A. (2005). Adoptive cell transfer therapy following non-myeloablative but lymphodepleting chemotherapy for the treatment of patients with refractory metastatic melanoma. J. Clin. Oncol..

[3] Chan J.D., Lai J., Slaney C.Y., Kallies A., Beavis P.A., Darcy P.K. (2021). Cellular networks controlling T cell persistence in adoptive cell therapy. Nat. Rev. Immunol..

[4] Figueroa D., Vega J.P., Hernández-Oliveras A., Ardiles F., Hidalgo S., López X., Saavedra V., Bustamante C., Malavé D., Flores F. (2026). Lymphodepleting preconditioning impairs host antitumor immunity induced by adoptive T cell therapy in mouse models. Nat. Commun..

[5] Spranger S., Dai D., Horton B., Gajewski T.F. (2017). Tumor-residing Batf3 dendritic cells are required for effector T cell trafficking and adoptive T cell therapy. Cancer Cell.

[6] Majzner R.G., Mackall C.L. (2018). Tumor antigen escape from CAR T-cell therapy. Cancer Discov..

[7] Walsh S.R., Simovic B., Chen L., Bastin D., Nguyen A., Stephenson K., Mandur T.S., Bramson J.L., Lichty B.D., Wan Y. (2019). Endogenous T cells prevent tumor immune escape following adoptive T cell therapy. J. Clin. Investig..

[8] Lauss M., Donia M., Harbst K., Andersen R., Mitra S., Rosengren F., Salim M., Vallon-Christersson J., Törngren T., Kvist A. (2017). Mutational and putative neoantigen load predict clinical benefit of adoptive T cell therapy in melanoma. Nat. Commun..

[9] Barras D., Ghisoni E., Chiffelle J., Orcurto A., Dagher J., Fahr N., Benedetti F., Crespo I., Grimm A.J., Morotti M. (2024). Response to tumor-infiltrating lymphocyte adoptive therapy is associated with preexisting CD8^+^ T-myeloid cell networks in melanoma. Sci. Immunol..

[10] Gattinoni L., Inchingolo G., Harrer D.C., Susana A., Puccio S., Slavkovic-Lukic D., Natrakul D.A., Strieder N., Heuser-Loy C., Baldwin J.G. (2026). Distinct in vivo dynamics of donor-derived stem cell memory CAR T cells post-allogeneic HSCT relapse. Cell.

